# Disappearance of peculiarly large atomic displacement in the high-temperature phase of magnetite with substitution of Fe^2+^ by Ni^2+^and Mn^2+^

**DOI:** 10.1107/S2052520625009928

**Published:** 2026-02-01

**Authors:** Takumi Kitamura, Takahiro Niimi, Hiroki Okudera

**Affiliations:** a Graduate School of Natural Science and Technology Kanazawa University Kakuma-machi Kanazawa Ishikawa920-1192 Japan; b School of Geosciences and Civil Engineering, College of Science and Engineering Kanazawa University Kakuma-machi Kanazawa Ishikawa920-1192 Japan; Polish Academy of Sciences, Poland

**Keywords:** magnetite, Fe_2_MnO_4_, Fe_2_NiO_4_, electron–phonon interaction

## Abstract

Single crystal specimens of Fe_2.992_O_4_ (magnetite), Fe_3–*x*–*y*_Mn*_x_*□*_y_*O_4_ (*x* ≤ 0.980) and Fe_3–*x*–*y*_Ni*_x_*□*_y_*O_4_ (*x* ≤ 0.513, □ denotes point defects) are prepared using a floating-zone technique, and changes in their structural parameter values with *x* are examined at room temperature. Preferences of Mn, Ni and point defects at A, B and B sites, respectively, are confirmed by single-crystal X-ray diffraction experiments, while preference of Mn at the A site is not perfectly accomplished in Fe_2.010_Mn_0.980_O_4_. Mean-square displacements of atoms along the 3 axis in [111] are peculiarly large at the B site in Fe_2.992_O_4_ and the displacement decreased smoothly in both series, to nearly half in Fe_2.010_Mn_0.980_O_4_, with increasing amount of heteroatom. On the other hand, displacements normal to the direction show only slight convexity with *x* due to coexistence of the heteroatom. As a result anisotropy in displacements (dominant in [111] at *x* = 0) is inverted at *x* = 0.3 in FeMn series and would expectedly be inverted at *x* ≃ 0.55 in FeNi series. In spite of different locations of heteroatoms and slightly different inversion points on anisotropy, changes in mean-square displacements at the B site in [111] in these series are found on similar lines with changing amounts of heteroatom. In other words, the amount of this displacement is a function of the amount of Fe^2+^, or remnant electron from the itinerant-electron point-of-view, on the B-site substructure in these compounds. This characteristic lattice mode on the B-site substructure could be interpreted as an average of local distortion of the substructure due to ‘trimeron’ [Senn *et al.* (2012). *Nature*, **481**, 173–176] and some other modes such as distortion in 〈001〉 [Siratori & Kino (1980). *J. Magn. Magn. Mat*. **20**, 87–90], which move in the high-temperature structure.

## Abstract 2

Structures of Fe_2.992_O_4_ (magnetite), Fe_3–*x*–*y*_Mn*_x_*□*_y_*O_4_ (*x* ≤ 0.980) and Fe_3–*x*–*y*_Ni*_x_*□*_y_*O_4_ (*x* ≤ 0.513, □ denotes point defects) are examined at room temperature. The observed disappearance of peculiarly large mean-square displacement at the B site along the 3 axis in [111] is interpreted as a disappearance of phonons interact with electrons in the high-temperature phase of magnetite.

## 1. Introduction

**Figure 1:**
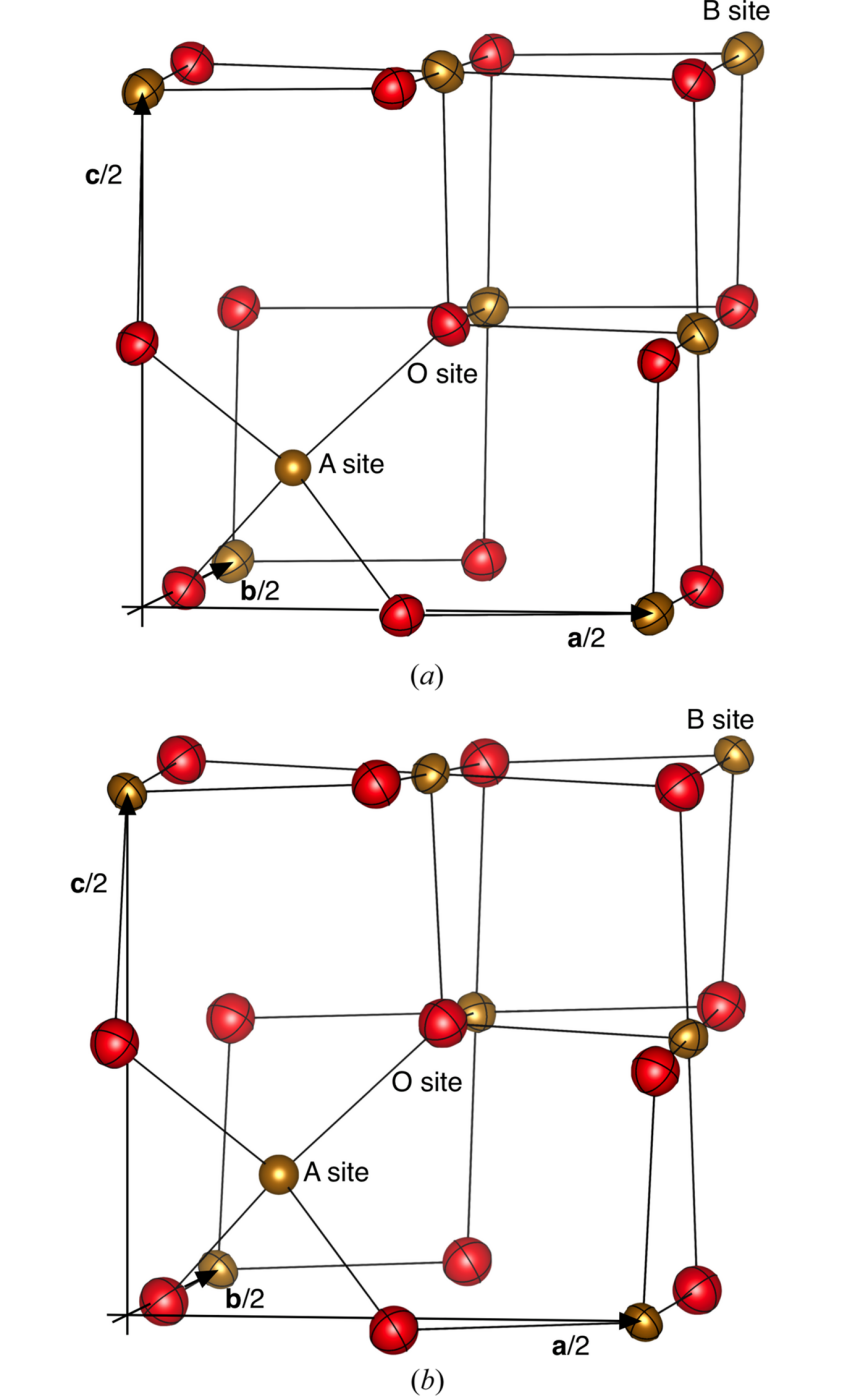
Structures of Fe_2.992_O_4_ (*a*) and Fe_2.010_Mn_0.980_O_4_ (*b*). Only 1/8 of the cells (−0.02 ≤ *x* ≤ 0.52, −0.02 ≤ *y* ≤ 0.52, −0.02 ≤ *z* ≤ 0.52) are shown with ADP ellipsoids of the 80% probability level. Brown sphere: A site; brown ellipsoid: B site; red ellipsoid: O site.

Studies on the crystal structure of magnetite (Fe_3_O_4_) have a long history since the room-temperature structure was solved in 1915 (Bragg, 1915 ▸; Nishikawa, 1915 ▸). There are numerous articles to date regarding relationships in its structure, physical properties such as magnetism and electrical conductivity, and the nature of semimetal–semiconductor phase transition (Verwey transition; Verwey & Haayman, 1941 ▸) at *T*_V_ ≃ 126 K. The high-temperature phase of magnetite crystallizes in the spinel-type structure (space group *Fd*3*m*) with structural-chemical formula ^iv^(Fe^3+^)^vi^[Fe^2+^_1/2_Fe^3+^_1/2_]_2_O_4_, where superscripts iv and vi denote tetrahedrally and octahedrally coordinated voids (A and B sites at Wyckoff positions 8*a* and 16*d*, respectively) in a cubic close-packing array of oxide anions (O sites at Wyckoff position 32*e*) (Fig. 1 ▸). While an ionic-crystal description is not adequate (*e.g*. Yanase & Siratori, 1984 ▸), the above description is widely accepted as a simplified picture of magnetite at temperatures above *T*_V_. On the other hand, the symmetry of its local structure has also been in debate: results of magnetocrystalline anisotropy measurements on the high-temperature phase (Siratori & Kino, 1980 ▸) first indicated the presence of local lattice distortion which did not obey the cubic symmetry.

One of the authors (HO) has reported the structure of magnetite in detail from 126 K (just above *T*_V_ of the specimen) up to 773 K (Okudera *et al.*, 1996 ▸). Apart from some anomalies at 126 K, there were two peculiarities on changes in structural parameters with temperature. One was a reversible change in the coordinate of the O site and the other was an inversion of anisotropy in the motion of Fe at the B site. The former had been explained as a gradual change in cation partitioning from ‘inverse’ to ‘random’ state at higher temperatures, and this interpretation was in agreement with thermal changes in the extraordinary Hall coefficient (Todo *et al.*, 1995 ▸) and thermoelectric power (Wu & Mason, 1981 ▸). On the other hand, no discussion was given for the latter in the article. At lower temperatures, the displacement of B-site Fe was so large along [111] that the displacement ellipsoid was prolate in the direction. This type of anisotropy itself is unique in magnetite among the oxide spinels. This anisotropy, however, reduced with increasing temperature. In other words, the displacement along [111] was less temperature dependent, and the anisotropy was inverted at ≃ 650 K. As a result, zero-point extrapolation of mean-square displacement (m.s.d., 〈*u*^2^〉 Å^2^) along [111] (0.006 Å^2^) was far larger than that for m.s.d.s normal to the direction (0.0017 Å^2^) and the experimentally determined value for octahedral cation sites at 10 K (0.0001–0.0017 Å^2^; Iizumi *et al.*, 1982 ▸). Since changes in the m.s.d.s were all smooth and linear, the m.s.d. in question was presumably raised by a constant amount of ≃ 0.004 Å^2^. The most intuitive explanation of this large m.s.d. was a splitting of the B-site position along [111] with disorder. If this is the case, there would be positive residuals on the 3 axis in the vicinity of the position after structure refinement even with anisotropic displacement parameters (ADPs). However, observed residual density in the region was negative and small (−0.3 e Å^−3^) (Okudera, 2000 ▸).

Atomic displacements obtained by X-ray investigation represent the convolution of all lattice vibrations, and we can not deconvolute them directly into individual lattice modes with amplitudes. However, high electrical conductivity of magnetite leads to an interpretation of this ‘raise’ of m.s.d. as a phonon which interacts with a conduction electron and thus is unique in the semimetallic phase. In reality, this anisotropy is apparently suppressed in cation-deficient specimens (Okudera, 1997 ▸). Prior to further considerations, the relationship between this large m.s.d. along [111] and electrical conductivity should be clarified for a series of compounds in which electrical conductivity has been measured. For this reason, the authors prepared Fe_3–*x*–*y*_Mn*_x_*□*_y_*O_4_ (*x* ≤ 0.980) and Fe_3–*x*–*y*_Ni*_x_*□*_y_*O_4_ (*x* ≤ 0.513) specimens (□ denotes point defects), and examined changes in the m.s.d. in question with composition. The anomaly on the m.s.d. in question will be discussed in relation to cooperative displacements of B-site Fe, which does not obey *Fd*3*m* symmetry but seen as a displacement of B-site Fe along [111] in the averaged cubic structure.

## 2. Experimental

### 2.1. Sample preparation and characterization

Fe_3–*x*–*y*_*D_x_*□*_y_*O_4_ (*D* = Mn and Ni) single crystals were grown using a floating zone technique in 〈110〉 at a growth rate of 5∼6 mm h^−1^ in CO_2_ atmosphere with a flow rate of 4.5 *l* min^−1^. The feed rods were prepared by mixing powdery α-Fe_2_O_3_ (95% as α-phase, Fujifilm Wako, Japan) and MnO_2_ (1st-grade, Kanto Chemical Co., Japan) or powdery Ni metal (99.99%, Fujifilm Wako) in target compositions (*x* = 0.1, 0.2, 0.3, 0.5, 0.6 and 1.0 for Mn, *x* = 0.1, 0.2, 0.3, 0.4, 0.5 and 0.6 for Ni). An Fe_3_O_4_ single crystal was also prepared. Hydro­statically pressed rods of the mixtures were calcined in air at 1100°C for 12 h. End-member Fe_3_O_4_ was grown first with natural magnetite of euhedral shape as a seed. The grown crystal of Fe_3_O_4_ was cut normal to the growth direction and used as seeds in the consecutive crystal growths including the Fe_3_O_4_ to be examined. Specimens will be abbreviated hereafter as the name of heteroatom with its content in the feed rods, such as Mn01 for Fe_2.9_Mn_0.1_O_4_ as a target composition, and as mgt#2 for Fe_3_O_4_ to be examined. Maximum Ni content in Fe_3–*x*–*y*_Ni*_x_*□*_y_*O_4_ was rather limited here since grown rods were polycrystalline when *x* > 0.6 and phase segregation with Fe-doped NiO occurred at *x* = 1.0.

Chemical compositions of the specimens were determined in two steps, (i) determination of the Fe/*D* ratio and (ii) evaluation of the cation deficiency. Grown crystals were halved along the growth direction for determination of Fe/*D* ratios. A Rigaku ZSX Primus II wavelength-dispersive spectrometer was used for X-ray fluorescence analyses with a Rh X-ray tube operated at 50 kV. Fe/*D* ratios were determined with calibration lines drawn with pure metal plates (99.99%) of Fe, Ni and Mn (Furuuchi Chemical Co., Japan) as standards. Firstly, a two-dimensional mapping of the ratios was carried out on the section plane to check if there was inhomogeneity in Fe/*D* ratio. For this purpose, a mask with a pinhole of 1.0 mm in diameter was inserted in between the X-ray source and the manoeuvrable sample stage. Fe/*D* maps showed that quenched molten zones at the top of grown crystals had slightly higher Mn or slightly lower Ni concentrations due to the different characters of these atoms on their compatibility to the solid phase. After that, the Fe/*D* ratio of each specimen used in the following sections was determined without the pinhole at the area where homogeneity was confirmed.

Actual compositions of grown crystals should be written as Fe_3–*x*–*y*_*D_x_*□*_y_*O_4_ since, as it has been pointed out by Kimura & Kitamura (1992 ▸), the growth operation was not at equilibrium and therefore the presence of cation deficiency was unavoidable on berthollide compounds. This non-equilibrium was enhanced (*y* increased) with decreasing temperature under a CO_2_ atmosphere. Therefore, *y* will be larger at the surface of the grown crystal closer to the seed crystal. From the considerations above, not only the round tip (quenched molten zone) but also exterior portions (≥ 0.5 mm from surface) of the grown crystals were trimmed out, and only portions of 2 mm from the top were used in the following examinations.

Amounts of cation deficiency were determined with thermogravimetry up to 800°C in air on a Rigaku ThermoPlus TG8120 thermal balance analyser except specimens Ni01 and Ni02. Temperature was kept at 800°C for 2 h to record a straight line on the profile after oxidation. Measurements were repeated twice for each specimen without sample exchange, and the profile of the second measurement was utilized to draw a baseline for the first profile. The *y* values on Ni-doped specimens tend to be less than those in Mn-doped ones as expected from their higher melting points. The *y* values of Ni01 and Ni02 were estimated by interpolation from those of mgt#2 and Ni03.

### 2.2. Data collection and unit-cell dimension determination

Specimens for X-ray diffraction experiments were taken from centre of the pellets, spherically ground (*d* = 0.10∼0.17 mm, mostly 0.14 mm) and mounted at the top of silica glass fibres of diameters of 0.08 mm with ep­oxy glue. The intensities of Bragg reflections and the values of θ were measured at room temperature using a Rigaku AFC-5S automated four-circle diffractometer with graphite-monochromatized Mo *Kα* radiation. The ω/2θ-scan mode was employed for the data collection, and other parameters such as scan width, offset and slit widths were optimized for each specimen. Unit-cell edge lengths were determined using 2θ values of eight peak positions of {8 8 8} reflections at 2θ ≃ 72° or those with 12 more peak positions of {0 8 8} at 2θ ≃ 57° and calibrated with Si (Okada & Tokumaru, 1984 ▸). See Tables 1 ▸, 2 ▸ and 3 ▸ for further details of specimens and data collection. Intensity data were converted to |*F*_obs_| and their standard uncertainties (s.u.s: σ), after applying Lorentz, polarization and spherical absorption corrections.

## 3. Structure refinements

### 3.1. Single O-site model

**Figure 2:**
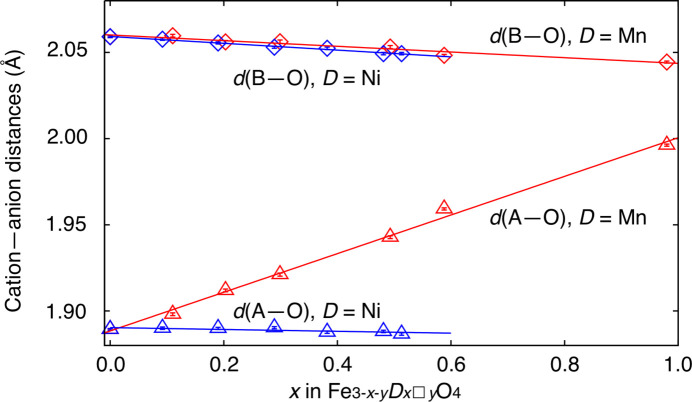
Changes in cation–anion distances with *x* after single O-site refinements. Triangles: *d*(A–O), diamonds: *d*(B–O). Red symbols and lines: FeMn series; blue symbols and lines: FeNi series. Linear regressions are shown as straight lines. Error bars are drawn inside symbols.

The least-squares program *LSGCEX* (Kihara, 1990 ▸) was used for structure refinements with variables including one scale and one isotropic extinction factor [type I with the Gaussian mosaic distribution of Becker & Coppens (1974 ▸)]. Averages over equivalent reflections were taken, and some weak [|
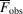
| < 3σ(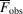)|] and less consistent (|*F*_obs_|_max_ ≥ 1.5 × |*F*_obs_|_min_ among equivalents) reflections were not used in least-squares cycles. The latter threshold was introduced to reduce the effect from simultaneous diffractions (Okudera *et al.*, 1996 ▸; Okudera, 2000 ▸) and the necessity of this will be discussed in §4.1. A simple weighting scheme with weights proportional to σ^−2^ was employed. Neutral form factors and their anomalous dispersion terms were taken from *International Tables for Crystallography*, Vol. C. The site occupancy at the O site was fixed to one. The refinements started from the coordinates and ADPs given for the stoichiometric magnetite specimen M104 given by Okudera (1997 ▸). Cation vacancy was assigned at the B site (Okudera, 1997 ▸). All Ni atoms were also assigned at the B site from results of magnetic moment measurement (Robertson & Pointon, 1966 ▸), Mössbauer spectroscopy (*e.g*. Sawatzky *et al.*, 1969 ▸; Sorescu *et al.*, 1998 ▸), EXAFS (Yao, 1992 ▸; Tangcharoen *et al.*, 2014 ▸) and single-crystal X-ray diffraction experiment utilizing anomalous dispersion effect (Tsukimura *et al.*, 1997 ▸). Partitioning of Mn over A and B sites in the FeMn series was controversial. Presence of Mn at both cation sites has been reported on Fe_2_MnO_4_ specimens with *i* = 0.1∼0.2, where *i* is a proportion of Mn at the B site to its total amount, with neutron diffractometry (Hastings & Corliss, 1956 ▸), Mössbauer spectroscopy (Sawatzky *et al.*, 1967 ▸; Topkaya *et al.*, 2016 ▸) and XAFS measurement (Tangcharoen *et al.*, 2014 ▸). On the other hand, examinations on specimens with lower Mn concentrations (*x* ≤ 0.54) commonly indicated the absence of Mn at the B site (Yadav *et al.*, 2015 ▸; Topkaya *et al.*, 2016 ▸; Okita *et al.*, 1998 ▸). However, Sorescu *et al.* (1998 ▸) and Varshney & Yogi (2011 ▸) proposed the sole occupation of Mn at the B site in Fe_3–*x*_Mn*_x_*O_4_ (*x* = 0.11 by Sorescu *et al.* and *x* = 0.10 and 0.50 by Varshney & Yogi) based on results of Mössbauer spectroscopy. Therefore, partitioning of Mn over A and B sites was examined on all data sets. When Mn was assigned at the A site and partitioning of the vacancy was refined, totals of site occupancies at cation sites fell within the range 0.983 (2)–0.997 (2) in Mn01–Mn06, indicating that the assignment of Mn at the A site was reasonable. Note that use of neutral form factors caused refined occupancies at cation sites smaller than real values in the stoichiometric specimen (Okudera, 1997 ▸). In contrast, refined totals of occupancies at A and B sites were 1.008 (2) and 0.991 (2), respectively, in Mn10 after the refinement with no restraint. This result suggested exchange of Mn, likely as Mn^3+^ (O’Handley, 2000 ▸), with Fe at the B site. When the vacancy was fixed at the B site and *i* was involved as varied parameter, calculation converged with *i* = 0.131 on Mn10. The *i* value went negative in Mn01–Mn06 under the same conditions and, therefore, Mn and vacancy were solely assigned at A and B sites, respectively, in consecutive iterations on Mn01–Mn06. The refined *i* value on Mn10 had to be a rough estimate for small difference in scattering powers of Fe and Mn and the value would be overestimated for use of neutral form factors, but the presence of Mn at the B site only in Mn10 was concordant within our data sets. To allow for this, here we assume the preference of Mn^2+^ at the A site and Mn^3+^ at the B site after oxidation of Fe^2+^ prior to Mn^2+^. Some Mn in the Mn10 specimen would be in the Mn^3+^ state for cation deficiency, and in this case the expected structural chemical formula is ^iv^(Fe^3+^_0.03_Mn^2+^_0.97_) ^vi^[Fe^3+^_1.98/2_Mn^3+^_0.01/2_]_2_ O_4_ and *i* = 0.01. While the precision of this structural chemical formula was rather limited, we refined the structure of specimen Mn10 with reference to this formula. Structural parameter values, m.s.d.s along principal axes and interatomic distances at this stage are listed in Tables 4 ▸ and 5 ▸. Selected interatomic distances and m.s.d.s of atoms at this stage are shown in Figs. 2 ▸ and 3 ▸, respectively.

### 3.2. Split O-site model for FeMn series

**Figure 3:**
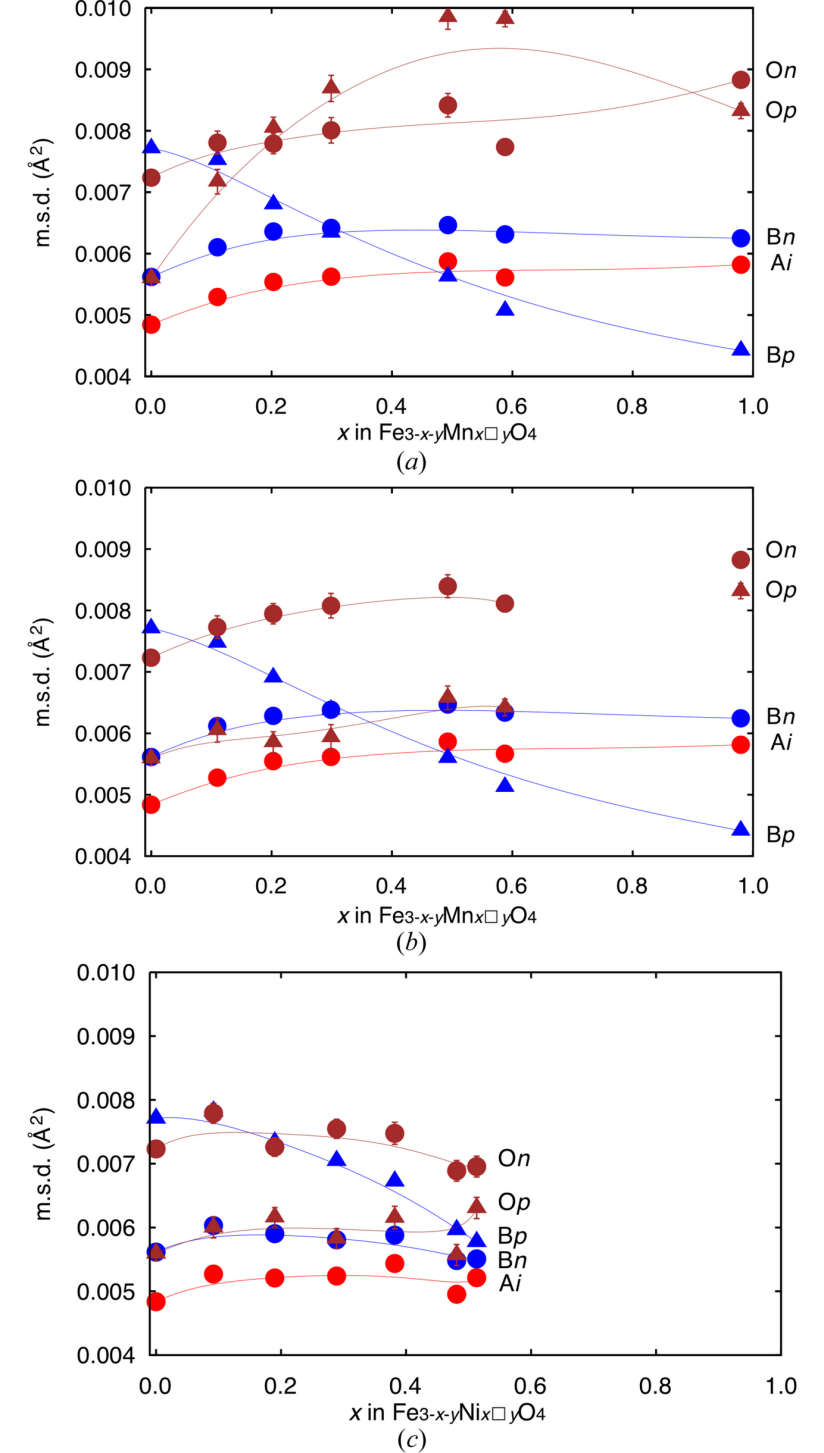
Changes in m.s.d.s (Å^2^) of atoms with *x* in principal axes of ADP ellipsoids in (*a*) FeMn series after single O-site refinements, (*b*) FeMn series after split O-site refinements and (*c*) FeNi series. Triangles: parallel to [111]; circles: normal to [111]. Red: A site; blue: B site; brown: O site. Some of the error bars are hidden behind symbols. Lines in respective colours are for guides for the eye.

Here we designate B and O with suffixes ‘*p*’ and ‘*n*’ for m.s.d.s in directions parallel and normal to [111] at respective sites, *e.g*. B*p* is the m.s.d. at the B site along [111]. Displacements are isotropic at the A site in harmonic analysis and suffix ‘*i*’ is added as the m.s.d. at the site. Not only on unit-cell edge lengths and cation–anion distances, mixing of cations affects refined ADP values too. This effect was apparent in the FeMn series as a linear increase of the A–O distance, *d*(A–O) (Å), as a function of *x* expressed by
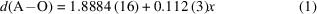
(Fig. 2 ▸) and a prominent convexity on change in O*p* [Fig. 3 ▸(*a*)]. We assumed substitution of (Fe^3+^O)_4_^5−^ by larger (Mn^2+^O)_4_^6−^ and further iterations were made with two oxide ion sites, O1 and O2 with *d*(A–O1) < *d*(A–O2) both at Wyckoff position 32*e*, with common ADP values. Occupancies at O1 and O2 sites were constrained to be equal to those of Fe and Mn, respectively, at the A site. Mn10 was not involved in this consideration for its small population of Fe at the A site. Positions of O1 and O2 sites were set to attain *d*(A–O1) = 1.888 Å and *d*(A–O2) = 2.000 Å from equation (1) by analogy with little change in *d*(A–O) in the FeNi series, in which the A site was solely occupied by Fe^3+^. The change in O*p* refined under distance-restraint is shown in Fig. 3 ▸(*b*) together with the other m.s.d. values, and now change in O*p* became concordant with that of A*i*. Note that A*i* and O*p* represent their displacements along their bond and should be close to each other when they were tightly bound. For this agreement, we took the split O-site model as the rational one for specimens Mn01–Mn06. The effect of Fe/Mn mixing over A and B sites in Mn10 was seen as far larger O*p* than A*i*, which was not obvious in the A–O distance. We did not apply the same consideration to the FeNi series since O*p* changed in harmony with A*i*. Summaries of structural analyses are given in Table 2 ▸. Refined ADPs and m.s.d.s of atoms are given in Table 6 ▸.

## 4. Results and discussion

### 4.1. Residual density

**Figure 4:**
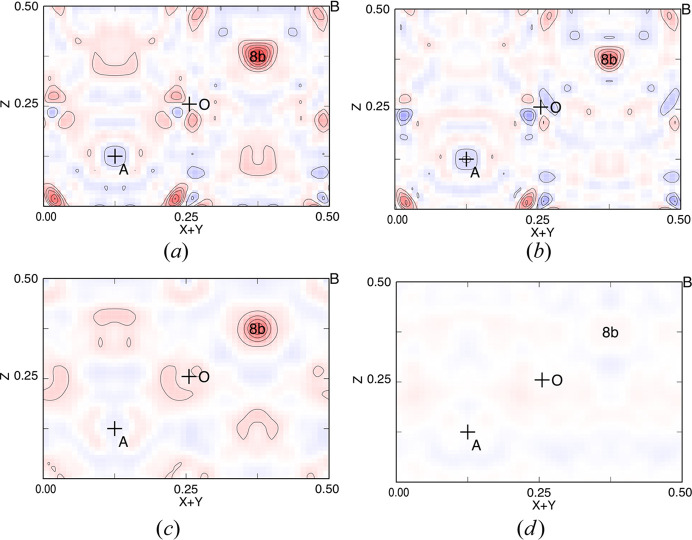
Residual density, Δρ, map after structure refinements of mgt#2 with different data truncation thresholds. Horizontal: **a** + **b**; vertical: **c**. Only 1/4 of the section for one cell is shown. Red region: positive; blue region: negative. Contours at every 0.5 e Å^−3^. Zero contours are omitted. Each of least-squares calculations employed reflections which obeyed the following conditions. (*a*) |
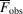
| ≥ 3σ(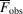) (common); *R*(*F*) = 0.022, *S* = 2.21 for 236 reflections. (*b*) 0.35 ≤ sinθ/λ (29.0° ≤ 2θ); *R*(*F*) = 0.021, *S* = 2.12 for 226 reflections. (*c*) |*F*_obs_|_max_ < 1.5 × |*F*_obs_|_min_ among equivalent reflections; *R*(*F*) = 0.0125, *S* = 1.91 for 190 reflections. (*d*) |*F*_obs_|_max_ < 1.05 × |*F*_obs_|_min_ among equivalent reflections; *R*(*F*) = 0.007, *S* = 1.01 for 79 reflections.

It is empirically known that residual density after structure refinement tends to be concentrated at special positions. In this study, prominent positive residuals over 2 e Å^−3^ appeared at Wyckoff position 8*b* (site symmetry 43*m* which is the highest site symmetry in the structure) in some structures. Other than random error on each |*F*_obs_| relating to counting statistics, there were two possible causes for the discrepancy between |
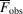
| and |*F*_calc_|. One is a misfit between assumed (neutral) and real atomic form factors in the low sinθ/λ region; in other words, the difference in spatial distribution of outer shell electrons of an atom between those after the RHF calculation and in the real crystal. Residual density (Δρ) maps after two refinements of mgt#2 structure, one with only |*F*_obs_| < 3σ(*F*_obs_) cutoff and the other with additional low-angle cutoff at sinθ/λ = 0.35 (2θ = 29.0°) are shown in Figs. 4 ▸(*a*) and 4 ▸(*b*), respectively. As it can be seen in the figures, there was no notable difference between these two maps, indicating that the discrepancies among |
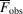
| with signs and *F*_calc_ occurred over the 2θ range examined. Another possible cause of these discrepancies was the occurrence of simultaneous diffraction (Cole *et al.*, 1962 ▸). This phenomenon causes apparent enhancement of weak, and a faint reduction of strong, diffraction intensities. Risk of ignoring this phenomenon has been pointed out by Fleet (1986 ▸) not only on space-group determination for false violation of extinction rules but also enhancement of weak diffraction intensities. This does not occur evenly among equivalent lattice points but some of those on routine data collection, and this phenomenon itself is not a unique issue on spinel phases but common on crystalline materials. Instead of examining contamination of intensities from simultaneously diffracted X-rays by repeating integration with multiple ψ angles at all reciprocal lattice points, yet another data truncation threshold based on the equivalence of observed structure amplitudes (eqvl) in equation |*F*_obs_|_max_ < eqvl × |*F*_obs_|_min_ among equivalent reflections was employed on structure refinement to select highly consistent and therefore expectedly less-contaminated |*F*_obs_|. Residual density maps after refinements of mgt#2 structure with |*F*_obs_| < 3σ(*F*_obs_) cutoff and different eqvl values are shown in Fig. 4 ▸. The residual density at position 8*b* diminished after refinement with the cutoff at eqvl = 1.05, whereas changes in refined parameter values with application of the cutoff were close to their combined s.u.s. This residual density would not diminish when there actually were interstitial atoms at that position since scattering power at the position should contribute to 72 out of 79 diffraction data used in the refinement. This relationship between eqvl value and residual density at position 8*b* had been confirmed in all datasets used in this study. Here we set eqvl = 1.5 to keep numbers of reflections secure to refine eight parameters. While residual density at position 8*b* was still high after these refinements (Tables 2 ▸ and 3 ▸), maximum positive densities in the vicinity of the B site [inside a box of 0.45 ≤ *x* (*y*, *z*) ≤ 0.55] were in the range 0.22 e Å^−3^ (Mn10) ∼ 0.61 e Å^−3^ (Ni06). No common feature was found on their appearance. Summarizing above, there was no sign of atoms located at positions other than A, B and O sites nor splitting of the B-site position in the present specimens.

### 4.2. Cation distribution and interatomic distances

Our single O-site refinements resulted in smooth changes of all cation–anion distances with increasing amounts of heteroatoms (Fig. 2 ▸). Their linear fashion indicated that they were refined as simple weighted averages of two (or more) bonds with different lengths. However, the values did not match the weighted averages of hitherto reported cation–anion distances.

As it has been pointed out, ionic radii considerations (Shannon, 1976 ▸) and bond-valence sum (BVS) (Brown & Altermatt, 1985 ▸) could not predict Fe–O separations in magnetite with precision, likely be due to its semimetallic nature (Okudera *et al.*, 1996 ▸). Observed *d*(A–O) [1.8893 (5) Å] and *d*(B–O) [2.0591 (5) Å] in mgt#2 did not match to those predicted from BVS with parameters by Brown & Altermatt [1.865 Å for *d*(^iv^Fe^3+^–O) and 2.078 Å as an average of *d*(^vi^Fe^3+^–O) = 2.015 Å and *d*(^vi^Fe^2+^–O) = 2.1405 Å]. These disagreements were not remedied with increasing amount of heteratoms. Extrapolation of *d*(B—O) at *x* = 1.0 was 2.040 Å for the FeNi series from linear regression of observations. This value is close to the average of predicted values for *d*(^vi^Fe^3+^–O) and *d*(^vi^Ni^2+^–O) (2.061 Å) from BVS, as if the electronic state of B-site Fe^3+^ would turn to that in ‘ionic’ compounds in Fe_2_NiO_4_. This agreement itself is in agreement with the Mössbauer spectrum on Fe_2.915_Ni_0.085_O_4_, which indicated the appearance of B-site Fe with more pronounced Fe^3+^ character by introducing Ni^2+^ (Sorescu *et al.*, 1998 ▸). However, extrapolated *d*(A–O) at *x* = 1.0 was 1.885 Å, and this is still far larger than the value for Fe^3+^ at the A site from BVS. Similarly, *d*(A–O) and *d*(B–O) in Mn10 were 1.9965 (6) Å and 2.0442 (6) Å, respectively. These values did not match weighted averages of predicted ones [2.040 Å and 2.015 Å, respectively, for *d*(A–O) and *d*(B–O) with *d*(^iv^Mn^2+^–O) = 2.046 Å and *d*(^vi^Mn^3+^–O) = 2.0165 Å from BVS]. Configuration at the A site to realize the observed distance with predicted *d*(^iv^Mn^3+^–O) = 1.8665 Å is ^iv^(Fe^3+^_0.03_Mn^2+^_0.245_Mn^3+^_0.725_). If this is the case, complementary configuration at the B site is ^vi^[Fe^2+^_0.362_Fe^3+^_0.627_Mn^3+^_0.005_] and predicted *d*(B–O) (2.061 Å) does not match the observation. These discrepancies indicated failure of a valence–distance relationship expected for ‘ionic’ compounds not only on magnetite but also in these series of compounds and this would conversely be the reason why Mössbauer spectroscopic studies successfully detected a sextet from the heteroatom of a distinct valence state. At least the change in O*p* in Mn01–Mn06 after split O-site refinements was smooth with reasonable values, supporting the relevance of the employed structure models with *d*(^iv^Fe—O1) = 1.888 Å and *d*(^iv^Mn—O2) = 2.000 Å.

### 4.3. Coordination polyhedra and ADPs

In the present specimens, refined ADPs involve local lattice distortion due to mixing of the heteroatom with different cation–anion separations. Observed weak convexity on changes in A*i* and B*n* (Fig. 3 ▸) reflected slight deviation in the position of each atom for the distortion. Changes in O*n* were in agreement with those of A*i* and B*n* in both series. B*p* decreased smoothly in both series with increasing amounts of heteroatom. As a result, B*p* reached its minimum value and became the smallest among m.s.d.s in Mn10 and so would be in Fe_2_NiO_4_ composition. Anisotropy in the displacement ellipsoid at the B site was inverted between prolate and oblate at *x* = 0.30 in the FeMn series and likely be inverted at *x* ≃ 0.55 in the FeNi series. In spite of the difference in inversion points, B*p* values in these two series were found on similar lines.

Success in structure refinements of FeMn series specimens with the split O-site model indicated virtually constant volumes of FeO_4_ and MnO_4_ tetrahedra in the FeMn series. The volume of FeO_4_ tetrahedra was smaller in the Ni06 specimen but their difference was marginal [3.4610 (16) Å^3^ in mgt#2 and 3.4458 (18) Å^3^ in Ni06]. The BO_6_ octahedron was elongated in [111] in a trigonal antiprismatic manner to shorten the shared edges with its neighbours in the structures. The quadratic elongation index and volume of the BO_6_ octahedron in mgt#2 were 1.0016 (4) and 11.613 (4) Å^3^, respectively. Volumes of BO_6_ octahedra reduced with increasing *x* in both series: calculated volumes were 11.444 (4) Å^3^ in Ni05 and 11.472 (6) Å^3^ in Mn05 after single-O site refinements. Deformations of BO_6_ octahedra, however, showed different trends in these series. The BO_6_ octahedron was further elongated in [111] with increasing Mn content. Quadratic elongation indices were 1.0039 (6) in Mn05 after the single-O site refinement and 1.0074 (4) in Mn10. On the other hand, the index in the Ni05 specimen was 1.0018 (5), indicating that incorporation of Ni at the B site reduced volume, but hardly changed shape of the BO_6_ trigonal antiprism.

From the viewpoint of steric repulsion, displacement of the B-site atom would be suppressed in [111] when the fractional coordinate of the O site, here referred to as *u* for historical reason, is larger than that in cubic closest packing and therefore the BO_6_ trigonal antiprism is elongated in [111]. This suppression will be stronger when *u* becomes larger. If this is the case, a relationship B*p* < B*n* would hold in these series over the range examined and B*p*/B*n* would be constant in the FeNi series. However, most of these predictions failed in these compounds: only the B*p* < B*n* relationship was found in the FeMn series specimens with *x* > 0.30. This relationship is also likely to be found in FeNi series specimens with *x* > 0.55, although we could not prepare such a specimen. Changes in interplanar separations of two basal planes (normal to [111]) of the antiprism also had no correlation with changes in B*p*. With reference to the results of single-O site refinements, the separation was increased from 2.472 Å in mgt#2 to 2.516 Å in Mn05 and decreased slightly to 2.467 Å in Ni05. It was hard to attribute inversion of anisotropy in ADPs at the B site, namely smooth and steady decrease of B*p*, to changes in their coordination environments.

### 4.4. Non-cubic distortion of the B-site substructure

**Figure 5:**
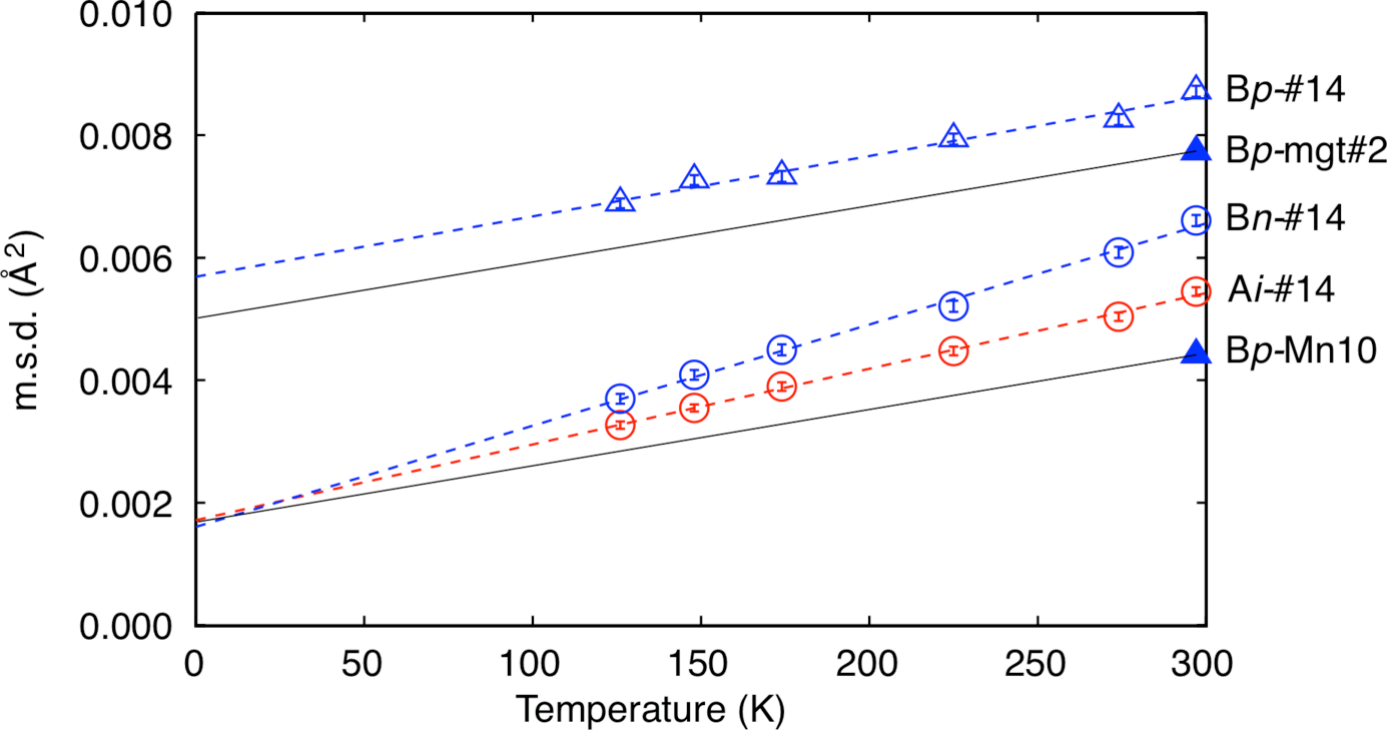
Selected m.s.d.s (Å^2^) in mgt#2, Mn10 and natural magnetite #14 (Okudera *et al.*, 1996 ▸) in heating cycle. Dashed lines in red and blue are linear regressions of observations. The thin black line starting from B*p*-mgt#2 was drawn as 88.5% of the regression line for B*p*-#14, and the line from B*p*-Mn10 was its parallel translation.

B*p* and B*n* in mgt#2 were, respectively, 0.00771 (5) Å^2^ and 0.00561 (4) Å^2^. In other words 88.5% and 84.7% of those in natural magnetite #14 at 297 K [B*p* = 0.00872 (9) Å^2^ and B*n* = 0.00661 (9) Å^2^] (Okudera *et al.*, 1996 ▸). Because these data sets were collected on the same diffractometer at the same temperature but were not analysed under identical calculation conditions, the data set of natural magnetite #14 at 297 K was re-analysed for comparison. Results of analysis showed that the effect of different extinction formalism was negligible. Half of the discrepancy was due to different assignments of cation vacancy in least-squares calculations. The remaining half of the discrepancy can be ascribed to impurities and encapsulation of specimen #14 in a silica glass capillary, which would cause more reduction of diffraction intensities on high-angle reflections measured at a χ angle closer to 90°. Fig. 5 ▸ shows changes in A*i*, B*p* and B*n* in natural magnetite #14 in the heating cycle together with selected m.s.d.s in mgt#2 and Mn10 for comparison. Extrapolated B*p* at 0 K from low-temperature data on natural magnetite #14 was approximately 0.0057 Å^2^ and the corresponding one for synthetic mgt#2 would be 0.0050 Å^2^ after correction. B*p* in Mn10 [0.00442 (4) Å^2^] was smaller than B*p* in mgt#2 and the difference was 0.0033 Å^2^. When this difference is applied at 0 K, B*p* in Mn10 at 0 K would be 0.0017 Å^2^. This value is fairly close to extrapolated A*i* and B*n* at 0 K from low-temperature data on natural magnetite #14 (0.0017 Å^2^ and 0.0016 Å^2^ for A*i* and B*n*, respectively). These consistencies indicate the presence of a lattice mode, which is unique in the high-temperature phase of magnetite and raise B*p* by a constant amount (0.0033 Å^2^ with reference to Mn10). This component was virtually temperature-independent, otherwise B*p* at 0 K would be negative (with negative dependence) or negative temperature dependence would occur on B*p* (with positive dependence) on Mn10, and this component was weakened as a function of increasing amounts of divalent heteroatom, here Ni^2+^ or Mn^2+^.

This high-temperature unique vibration mode on the B-site substructure could be a convolution of multiple modes averaged under cubic symmetry. While recent advancements on understanding of the low-temperature structure suggested the presence of a lot of frozen lattice modes in the low-temperature structure (Attfield, 2014, ▸ and references therein), a simple interpretation of this upward shift is the observation of a ‘trimeron’, namely, correlated stretch/shrink of a trimer made of neighbouring three B-site Fe atoms arrayed in 〈110〉 (cubic setting) (Senn *et al.*, 2012 ▸). This B-site trimer was detected from the splitting of diffraction spots at temperatures below *T*_V_ and considered as one of the frozen phonon modes. A similar idea was first proposed by Yamada *et al.* (1979 ▸) as a ‘molecular polaron’, namely, correlated displacements of atoms to describe charge density fluctuation in the cubic phase, although they attributed the shifts at O sites. This mode does not follow cubic symmetry, and this stretch/shrink in three directions around the 3 axis can be seen as displacement of the B-site cations in [111] when the structure is refined under cubic symmetry. Expected local distortion on the B-site substructure from this mode follows two out of three directions in the scheme proposed by Siratori & Kino (1980 ▸). However, a trimeron would not be the only mode which raised B*p*. As mentioned in §4.1, positive residual densities in the vicinity of the B-site position are not particularly high on 〈110〉. B-site Fe seemed to move also in other directions such as 〈001〉, which is the one remaining direction in the distortion scheme of Siratori & Kino (1980 ▸).

Our observations indicated that those temperature-independent components run across the structure in the high-temperature phase of magnetite with combined amplitude large enough to be seen as an upward-shift of B*p*. Decrease of B*p* with *x* was smooth and linear, like changes in electrical conductivities with *x* in Fe_3–*x*_Ni*_x_*O_4_ (Whall *et al.*, 1986 ▸) and Fe_3–*x*_Mn*_x_*O_4_ (Phillips *et al.*, 1995 ▸). The number of Fe^2+^ at the B site in the present specimens also has an inverse relationship with *x*. Therefore, peculiar behaviour on B*p*, the number of remnant electrons in the B-site substructure from an itinerant electron viewpoint, and electrical conductivity in these series could be connected *via* phonon coupled with electron transport. Due to limitations of X-ray diffraction experiments we did not look in detail at the electron–phonon interaction and conduction mechanism, such as a dynamical nature of trimeron in the high-temperature structure. However, we succeeded in showing the relationship between the peculiarly large B*p* and uniqueness in the physical properties of the high-temperature phase of magnetite with a high degree of consistency.

## Supplementary Material

Crystal structure: contains datablock(s) mgt2, mn01, mn02, mn03, mn05, mn06, mn10, ni01, ni02, ni03, ni04, ni05, ni06. DOI: 10.1107/S2052520625009928/lo5130sup1.cif

Structure factors: contains datablock(s) mgt2. DOI: 10.1107/S2052520625009928/lo5130mgt2sup2.hkl

Structure factors: contains datablock(s) mn01. DOI: 10.1107/S2052520625009928/lo5130mn01sup3.hkl

Structure factors: contains datablock(s) mn02. DOI: 10.1107/S2052520625009928/lo5130mn02sup4.hkl

Structure factors: contains datablock(s) mn03. DOI: 10.1107/S2052520625009928/lo5130mn03sup5.hkl

Structure factors: contains datablock(s) mn05. DOI: 10.1107/S2052520625009928/lo5130mn05sup6.hkl

Structure factors: contains datablock(s) mn06. DOI: 10.1107/S2052520625009928/lo5130mn06sup7.hkl

Structure factors: contains datablock(s) mn10. DOI: 10.1107/S2052520625009928/lo5130mn10sup8.hkl

Structure factors: contains datablock(s) ni01. DOI: 10.1107/S2052520625009928/lo5130ni01sup9.hkl

Structure factors: contains datablock(s) ni02. DOI: 10.1107/S2052520625009928/lo5130ni02sup10.hkl

Structure factors: contains datablock(s) ni03. DOI: 10.1107/S2052520625009928/lo5130ni03sup11.hkl

Structure factors: contains datablock(s) ni04. DOI: 10.1107/S2052520625009928/lo5130ni04sup12.hkl

Structure factors: contains datablock(s) ni05. DOI: 10.1107/S2052520625009928/lo5130ni05sup13.hkl

Structure factors: contains datablock(s) ni06. DOI: 10.1107/S2052520625009928/lo5130ni06sup14.hkl

CCDC references: 2501894, 2501895, 2501896, 2501897, 2501898, 2501899, 2501900, 2501901, 2501902, 2501903, 2501904, 2501905, 2501906

## Figures and Tables

**Table 1:** Common crystallographic data and data collection conditions

Crystal data	
Crystal system	Cubic
Space group	*Fd*3*m* (No. 227, origin choice 2)
*Z*	8
Crystal shape	Sphere

Data collection	
Radiation type	Mo *K*α
Wavelength (Å)	0.71069
Diffractometer	Rigaku AFC-5S
Data collection method	ω–2θ scan
Scan speed (° min^−1^)	4
2θ (°) collected	≤ 100 (120 on mgt#2)
Reciprocal space	*h* ≥ 0, *k* ≥ 0, *l* ≥ 0 and their Friedel pairs

Refinement	
Data truncation criteria	| 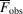 | ≥ 3σ( 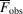 ), |*F*_obs_|_max_ < 1.5|*F*_obs_|_min_ among equivalents
Refinement method	Full-matrix least-squares on *F*
Extinction formalism	Becker & Coppens (1974 ▸) type 1 Gaussian, isotropic

**Table 2:** Details of crystallographic data, data collection and structure refinements for Fe_3–*x*–*y*_Mn*_x_*□*_y_*O_4_

	mgt#2	Mn01	Mn02	Mn03	Mn05	Mn06	Mn10
Crystal data							
Formula	Fe_2.992_□_0.008_O_4_	Fe_2.865_Mn_0.110_□_0.025_O_4_	Fe_2.771_Mn_0.203_□_0.026_O_4_	Fe_2.666_Mn_0.299_□_0.035_O_4_	Fe_2.473_Mn_0.493_□_0.034_O_4_	Fe_2.397_Mn_0.588_□_0.015_O_4_	Fe_2.010_Mn_0.980_□_0.010_O_4_
*a* (Å)	8.3975 (2)	8.4122 (4)	8.4232 (4)	8.4370 (6)	8.4606 (5)	8.4718 (6)	8.5149 (3)
Volume (Å^3^)	592.17 (4)	595.29 (8)	597.63 (9)	600.57 (13)	605.62 (11)	608.03 (13)	617.36 (7)
*D_x_* (g cm^−3^)	5.184	5.133	5.110	5.072	5.027	5.029	4.951
*F*(000)	878.336	873.920	872.968	870.328	868.984	872.176	870.080
μ (mm^−1^)	14.267	14.045	13.928	13.758	13.528	13.503	13.084
Crystal size (diameter, mm)	0.18	0.10	0.17	0.12	0.10	0.14	0.17
μ*r*	1.2840	0.7023	1.1839	0.8255	0.6764	0.9452	1.1121

Data collection							
Reciprocal space (maximum Laue index)	20	18	18	18	18	18	18
No. of measured, independent and used reflections	2314, 257, 190	1580, 182, 127	1598, 184, 137	1616, 186, 135	1616, 186, 136	1616, 186, 143	1628, 187, 153
*R*_int_ (%) for used reflections	1.44	2.03	1.38	1.67	1.87	1.53	1.39

Refinement							
Single O-site model							
No. of parameters	8	8	8	8	8	8	8
*R*(*F*), *R*(*F*^2^), w*R*(*F*)	0.013, 0.033, 0.017	0.011, 0.022, 0.014	0.013, 0.029, 0.016	0.013, 0.026, 0.017	0.011, 0.020, 0.015	0.009, 0.022, 0.012	0.012, 0.028, 0.015
*S*(*F*)	1.906	1.207	1.659	1.700	1.363	1.218	1.532
Δρ_max_, Δρ_min_ (e Å^−3^)	2.38, −0.49	0.83, −0.64	1.82, −0.56	1.72, −0.92	1.65, −0.96	1.40, −0.48	1.73, −0.56
Δρ_max_ (e Å^−3^) in vicinity of B-site position	0.470	0.335	0.375	0.412	0.303	0.246	0.122

Split O-site model (pos.fix)							
No. of parameters		7	7	7	7	7	
*R*(*F*), *R*(*F*^2^), w*R*(*F*)		0.011, 0.021, 0.014	0.013, 0.030, 0.016	0.013, 0.026, 0.018	0.011, 0.020, 0.015	0.011, 0.023, 0.013	
*S*(*F*)		1.202	1.677	1.699	1.359	1.333	
Δρ_max_, Δρ_min_ (e Å^−3^)		0.79, −0.63	1.96, −0.67	1.75, −0.91	1.62, −0.96	1.49, −0.60	
Δρ_max_ (e Å^−3^) in vicinity of B-site position		0.332	0.395	0.425	0.300	0.295	

**Table 3:** Details of crystallographic data, data collection and structure refinements for Fe_3–*x*–*y*_Ni*_x_*□*_y_*O_4_

	Ni01	Ni02	Ni03	Ni04	Ni05	Ni06
Formula	Fe_2.902_Ni_0.092_□_0.006_O_4_†	Fe_2.805_Ni_0.190_□_0.005_O_4_†	Fe_2.708_Ni_0.289_□_0.003_O_4_	Fe_2.608_Ni_0.382_□_0.010_O_4_	Fe_2.514_Ni_0.481_□_0.005_O_4_	Fe_2.462_Ni_0.513_□_0.025_O_4_
*a* (Å)	8.3948 (5)	8.3886 (4)	8.3826 (6)	8.3770 (4)	8.3692 (3)	8.3669 (10)
Volume (Å^3^)	591.60 (11)	590.29 (8)	589.03 (12)	587.85 (8)	586.21 (6)	585.7 (2)
*D_x_* (g cm^−3^)	5.197	5.217	5.237	5.244	5.272	5.253
*F*(000)	880.224	882.000	884.000	884.032	886.656	883.008
μ (mm^−1^)	14.425	14.609	14.794	14.928	15.140	15.105
Crystal size (diameter, mm)	0.12	0.14	0.14	0.14	0.16	0.14
μ*r*	0.866	1.023	1.036	1.045	1.211	1.057

Data collection						
Reciprocal space (maximum Laue index)	18	18	17	17	17	17
No. of measured, independent and used reflections	1580, 182, 129	1580, 182, 131	1562, 180, 137	1562, 180, 136	1562, 180, 139	1562, 180, 140
*R*_int_ (%) for used reflections	1.49	1.46	1.31	1.46	1.27	1.39

Refinement						
Single O-site model						
No. of parameters	8	8	8	8	8	8
*R*(*F*), *R*(*F*^2^), w*R*(*F*)	0.010, 0.021, 0.013	0.011, 0.027, 0.013	0.013, 0.033, 0.015	0.013, 0.026, 0.017	0.014, 0.036, 0.017	0.014, 0.036, 0.017
*S*(*F*)	1.296	1.387	1.534	1.734	1.876	1.876
Δρ_max_, Δρ_min_ (e Å^−3^)	1.17, −0.49	1.01, −0.41	1.52, −0.48	1.78, −0.78	2.04, −0.47	1.90, −0.66
Δρ_max_ (e Å^−3^) in vicinity of B-site position	0.250	0.301	0.377	0.414	0.480	0.613

† Estimated. See text.

**Table:** *x* = *y* = *z*, *U*_11_ = *U*_22_ = *U*_33_ and *U*_12_ = *U*_13_ = *U*_23_ at all atomic sites. *U*_12_ = 0 at the A site.

		mgt#2	Mn01	Mn02	Mn03	Mn05	Mn06	Mn10
Extinction factor		0.282 (10)	0.209 (9)	0.244 (9)	0.200 (11)	0.210 (10)	0.203 (7)	0.170 (8)
Site, site symmetry								
A, 43*m*	*x*	1/8	1/8	1/8	1/8	1/8	1/8	1/8
	Occ. Fe	1	0.8900	0.7970	0.7010	0.5070	0.4120	0.030
	Occ. Mn	–	0.1100	0.2030	0.2990	0.4930	0.5880	0.970
	*U*_11_ = A*i*	0.00484 (5)	0.00529 (6)	0.00553 (7)	0.00562 (8)	0.00587 (7)	0.00561 (5)	0.00568 (6)
B, 3*m*.	*x*	1/2	1/2	1/2	1/2	1/2	1/2	1/2
	Occ. Fe	0.9960	0.9875	0.9870	0.9825	0.9830	0.9925	0.990
	Occ. Mn	–	0	0	0	0	0	0.005
	*U*_11_	0.00631 (5)	0.00657 (7)	0.00650 (7)	0.00639 (8)	0.00618 (7)	0.00589 (5)	0.00564 (6)
	*U*_12_	0.00070 (4)	0.00047 (7)	0.00015 (5)	−0.00003 (6)	−0.00028 (5)	−0.00041 (4)	−0.00061 (3)
	B*p*	0.00771 (4)	0.00752 (7)	0.00680 (6)	0.00634 (7)	0.00562 (6)	0.00507 (4)	0.00442 (4)
	B*n*	0.00561 (4)	0.00610 (7)	0.00636 (6)	0.00641 (7)	0.00646 (6)	0.00631 (4)	0.00626 (5)
O, 3*m*.	*x*	0.25490 (6)	0.25527 (9)	0.25605 (9)	0.25646 (11)	0.25757 (10)	0.25852 (7)	0.26037 (7)
	*U*_11_	0.00668 (10)	0.00759 (14)	0.00787 (15)	0.00823 (18)	0.00889 (17)	0.00842 (12)	0.00857 (14)
	*U*_12_	−0.00055 (13)	−0.0002 (2)	0.00009 (18)	0.0002 (2)	0.0005 (2)	0.00069 (13)	−0.00019 (12)
	O*p*	0.00559 (12)	0.0072 (2)	0.00804 (17)	0.0087 (2)	0.0098 (2)	0.00981 (13)	0.00818 (13)
	O*n*	0.00722 (11)	0.00780 (19)	0.00779 (17)	0.0080 (2)	0.00841 (19)	0.00773 (13)	0.00876 (13)

**Table:** 

		Ni01	Ni02	Ni03	Ni04	Ni05	Ni06
Extinction factor		0.193 (8)	0.264 (9)	0.310 (10)	0.180 (9)	0.284 (11)	0.337 (13)
Site, site symmetry							
A, 43*m*	*x*	1/8	1/8	1/8	1/8	1/8	1/8
	Occ. Fe	1	1	1	1	1	1
	*U*_11_ = A*i*	0.00527 (6)	0.00521 (6)	0.00524 (7)	0.00544 (7)	0.00495 (7)	0.00521 (7)
B, 3*m*.	*X*	1/2	1/2	1/2	1/2	1/2	1/2
	Occ. Fe	0.9510	0.9027	0.8540	0.8040	0.7570	0.7310
	Occ. Ni	0.0459	0.0950	0.1445	0.1910	0.2405	0.2565
	*U*_11_	0.00663 (6)	0.00638 (6)	0.0622 (7)	0.00616 (7)	0.00564 (7)	0.00560 (7)
	*U*_12_	0.00060 (6)	0.00048 (5)	0.00041 (5)	0.00028 (5)	0.00016 (5)	0.00009 (5)
	B*p*	0.00782 (6)	0.00734 (5)	0.00705 (5)	0.00672 (6)	0.00596 (6)	0.00577 (6)
	B*n*	0.00603 (6)	0.00590 (6)	0.00581 (6)	0.00588 (6)	0.00548 (6)	0.00551 (6)
O, 3*m*.	*x*	0.25499 (8)	0.25507 (7)	0.25520 (7)	0.25510 (8)	0.25525 (8)	0.25518 (8)
	*U*_11_	0.00719 (13)	0.00689 (12)	0.00697 (14)	0.00704 (16)	0.00645 (15)	0.00674 (15)
	*U*_12_	−0.00060 (18)	−0.00037 (16)	−0.00057 (16)	−0.00044 (19)	−0.00044 (17)	−0.00022 (17)
	O*p*	0.00600 (17)	0.00616 (15)	0.00583 (16)	0.00615 (18)	0.00557 (17)	0.00631 (17)
	O*n*	0.00779 (16)	0.00726 (15)	0.00755 (15)	0.00747 (17)	0.00689 (16)	0.00696 (16)

**Table:** 

	mgt#2	Mn01	Mn02	Mn03	Mn05	Mn06	Mn10
A site							
A–O (×4)	1.8893 (5)	1.8981 (8)	1.9119 (8)	1.9211 (9)	1.9428 (8)	1.9592 (6)	1.9965 (6)
Polyhedral volume	3.4610 (16)	3.509 (2)	3.587 (2)	3.639 (3)	3.763 (2)	3.8595 (18)	4.0841 (18)
B site							
B–O (×6)	2.0591 (5)	2.0597 (8)	2.0561 (8)	2.0562 (10)	2.0531 (9)	2.0483 (6)	2.0442 (6)
Polyhedral volume	11.613 (4)	11.618 (5)	11.547 (5)	11.543 (6)	11.472 (6)	11.374 (4)	11.265 (4)
Quadratic elongation	1.0016 (4)	1.0018 (5)	1.0024 (5)	1.0028 (6)	1.0039 (6)	1.0049 (4)	1.0074 (4)
Separation between two pinacoids	2.4716 (6)	2.4796 (9)	2.4904 (9)	2.4985 (11)	2.5164 (10)	2.5289 (7)	2.5600 (7)

**Table:** 

	Ni01	Ni02	Ni03	Ni04	Ni05	Ni06
A site						
A–O (×4)	1.8900 (7)	1.8899 (6)	1.8903 (6)	1.8877 (7)	1.8881 (7)	1.8866 (7)
Polyhedral volume	3.4650 (19)	3.4640 (17)	3.4666 (16)	3.4523 (11)	3.4541 (18)	3.4458 (18)
B site						
B–O (×6)	2.0577 (7)	2.0555 (6)	2.0530 (6)	2.0524 (7)	2.0493 (7)	2.0493 (7)
Polyhedral volume	11.588 (5)	11.550 (4)	11.507 (4)	11.497 (5)	11.444 (4)	11.445 (4)
Quadratic elongation	1.0017 (5)	1.0017 (4)	1.0018 (4)	1.0017 (5)	1.0018 (5)	1.0018 (4)
Separation between two pinacoids	2.4717 (8)	2.4707 (7)	2.4702 (7)	2.4676 (8)	2.4667 (8)	2.4654 (8)

**Table 6:** Extinction factor, atomic coordinates, anisotropic displacement parameters (Å^2^) and mean-square displacements of atoms (Å^2^) after split O-site refinements *x* = *y* = *z*, *U*_11_ = *U*_22_ = *U*_33_ and *U*_12_ = *U*_13_ = *U*_23_ at all atomic sites. *U*_12_ = 0 at the A site. Common *U*s were assigned to O1 and O2. Occupancies at O1 and O2 were set identical with those of Fe and Mn, respectively, at the A site.

		Mn01	Mn02	Mn03	Mn05	Mn06
Extinction factor		0.208 (9)	0.245 (9)	0.200 (11)	0.210 (10)	0.204 (8)
Site, site symmetry						
A, 43*m*	*x*	1/8	1/8	1/8	1/8	1/8
	Occ. Fe	0.8900	0.7970	0.7010	0.5070	0.4120
	Occ. Mn	0.1100	0.2030	0.2990	0.4930	0.5880
	*U*_11_ = A*i*	0.00528 (7)	0.00555 (7)	0.00561 (8)	0.00586 (7)	0.00567 (6)
B, 3*m*.	*x*	1/2	1/2	1/2	1/2	1/2
	Occ. Fe	0.9875	0.9870	0.9825	0.9830	0.9925
	Occ. Mn	0	0	0	0	0
	*U*_11_	0.00657 (7)	0.00649 (7)	0.00638 (8)	0.00618 (7)	0.00593 (5)
	*U*_12_	0.00045 (6)	0.00021 (5)	0.00000 (5)	−0.00029 (5)	−0.00040 (4)
	B*p*	0.00748 (5)	0.00691 (6)	0.00638 (8)	0.00560 (6)	0.00513 (4)
	B*n*	0.00612 (7)	0.00628 (6)	0.00638 (8)	0.00647 (6)	0.00634 (5)
O1, 3*m*.	*x*	0.254585	0.254416	0.254204	0.253844	0.253673
O2, 3*m*.	*x*	0.262279	0.262099	0.261875	0.261493	0.261313
	*U*_11_	0.00717 (15)	0.00725 (15)	0.00736 (18)	0.00779 (17)	0.00755 (13)
	*U*_12_	−0.0006 (2)	−0.00069 (18)	−0.0007 (2)	−0.0006 (2)	−0.00056 (14)
	O*p*	0.0061 (2)	0.00587 (17)	0.0059 (2)	0.00658 (19)	0.00642 (14)
	O*n*	0.00773 (18)	0.00795 (17)	0.0081 (2)	0.00840 (19)	0.00811 (14)
